# Short-term prophylaxis (STP) with plasma derived human C1 inhibitor concentrate (pdhC1-INH) in two pregnant women with hereditary angioedema (HAE): an experience in Rio de Janeiro – Brazil

**DOI:** 10.1186/1939-4551-8-S1-A127

**Published:** 2015-04-08

**Authors:** Solange Oliveira Rodrigues Valle, Maria Luiza Oliva Alonso, Sérgio Duarte Dortas, Soloni Afra Pires Levy, Ana Paula Ferracciú Coutinho Millet, Alfeu Tavares França, Alfeu Tavares França, Ana Luiza Ribeiro Bard De Carvalho

**Affiliations:** 1 Hospital Universitário Clementino Fraga Filho Hucff-Ufrj, Brazil; 2 Universidade Iguaçu, Brazil; 3 Hospital São Zacharias, Brazil

## Background

HAE is an inherited disease characterized by sudden, recurrent episodes of edema involving the skin, gastrointestinal, respiratory tract and other organs. Pregnancy can mitigate, aggravate or have no effect on HAE C1-INH edematous attacks. Short term prophylaxis is recommended before labor and delivery when HAE C1-INH symptoms have recurred frequently during the third trimester of pregnancy. The administration of pdhC1-INH in HAE is recommended as the first line therapy in pregnancy. It is effective and safe. However, pdhC1-INH is not available in many countries, such as in Brazil. In these cases fresh frozen plasma might serve as an alternative for STP (evidence level III). We describe our experience with pdh C1-INH in two pregnant patients with HAE followed up in a Reference Center in Rio de Janeiro, who received the medicine by means of Justice.

## Methods

CASE 1- DFT, a 29-year-old pregnant woman, with HAE Type I (C4 = 5,0 mg/dL e C1-INH = 9,0 mg/dL) and recurrent edema of hands, feets, lips, larynge and abdominal pain. She received 1000 units of pdh C1-INH, intravenously (IV), on the day of delivery. CASE 2 - RSS, a 30 year-old pregnant woman, with HAE Type I (C4 = 5,0 mg/dL e C1-INH = 5,0 mg/dL) and episodes of swelling in the hands, feet and abdominal pain. She also received 1000 units of pdh C1-INH, IV, on the day of delivery.

## Results

Both of them had uncomplicated labor under pdh C1-INH prophylaxis. Healthy infants were born.

## Conclusions

Our patients could experience uncomplicated labors while being administered prophylactic pdhC1-INH, despite having some attacks of HAE during pregnancy. Short-term prophylaxis is important in individuals with known HAE who are undergoing procedures which can potentially precipitate an attack, as labor. pdhC1-INH concentrate should be always available to be used, if necessary.

## Consent

Written informed consent was obtained from the patient for publication of this abstract and any accompanying images. A copy of the written consent is available for review by the Editor of this journal.

